# Holistic Approach to the Diagnosis and Treatment of Patients with Tumor Metastases to the Spine

**DOI:** 10.3390/cancers14143480

**Published:** 2022-07-18

**Authors:** Hanna Nowak, Dominika Maria Szwacka, Monika Pater, Wojciech Krzysztof Mrugalski, Michał Grzegorz Milczarek, Magdalena Staniszewska, Roman Jankowski, Anna-Maria Barciszewska

**Affiliations:** 1 Medical Faculty, Karol Marcinkowski University of Medical Sciences, Fredry 10, 61-701 Poznan, Poland; dominikaszwacka@gmail.com (D.M.S.); monika.pater98@gmail.com (M.P.); mrugalskiwojtek@gmail.com (W.K.M.); michal.milczarek2@onet.pl (M.G.M.); m.stanishewska@gmail.com (M.S.); 2 Chair and Department of Neurosurgery and Neurotraumatology, Karol Marcinkowski University of Medical Sciences, Przybyszewskiego 49, 60-355 Poznan, Poland; barciszewska.anna@spsk2.pl; 3 Department of Neurosurgery and Neurotraumatology, Heliodor Swiecicki Clinical Hospital, Przybyszewskiego 49, 60-355 Poznan, Poland; 4 Intraoperative Imaging Unit, Chair and Department of Neurosurgery and Neurotraumatology, Karol Marcinkowski University of Medical Sciences, Przybyszewskiego 49, 60-355 Poznan, Poland

**Keywords:** metastases, spine, quality of life, scales, surgical treatment, decompression, tumor

## Abstract

**Simple Summary:**

Spine metastases constitute a significant social problem. The spine is a frequent site of metastases of various neoplasms. Our goal was to draw attention to the complexity of that problem and the diagnostic possibilities, qualification nuances, and application of the appropriate treatment method. We focused on the holistic approach to patient care. This review comprehensively covers the current knowledge in the field.

**Abstract:**

The treatment of neoplastic spine metastases requires multi-faceted assessment and an interdisciplinary approach to patients. The metastases do not show specific symptoms but are often the first confirmation of the presence of a primary tumor in a patient. The diagnostic process includes imaging and invasive procedures, e.g., biopsy. It is essential to qualify the patient for an appropriate treatment using dedicated scales. Decompression of the spinal cord is a critical issue to save or restore neurological function in a patient with spine metastases. Surgical treatment ought to meet three criteria: release spinal cord and nerve roots, restore the spine’s anatomical relations, and ensure the internal stabilization of the spine. A good result from surgical treatment enables the continuation of radiotherapy, chemotherapy, hormone therapy, and targeted molecular therapy. Stereotactic radiosurgery and stereotactic body radiotherapy are more effective ways of treating spine metastases than conventional external beam radiotherapy. They allow higher doses of radiation, concentrated precisely at the tumor site. Our review summarizes the established and emerging concepts in the treatment of spine metastases. A holistic approach to the patient enables the selection of the appropriate therapy.

## 1. Introduction

**Figure 1:**
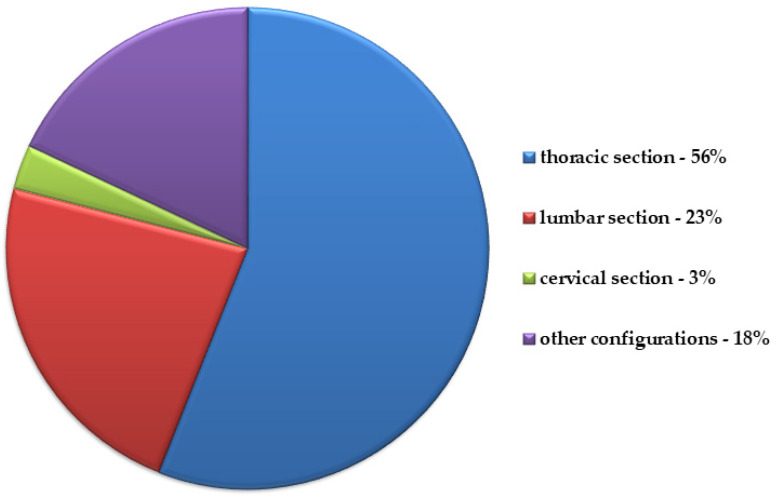
The frequency of metastases in individual sections of the spine based on data from ref. [6].

Metastases to the spine are much more frequent than primary spine tumors [1,2]. A significant amount of patients with generalized neoplastic disease develop bone metastases: about 6.9–8.6% [3,4]. The skeletal system is the third most frequent location of neoplastic metastases after the lungs and liver [5]. Although bone metastases are an expression of a significant advancement of the neoplastic process, it is sometimes the first and only symptom of a neoplastic disease that manifests itself clinically or radiologically [5,6]. If the metastasis to the bones is a secondary symptom of neoplastic disease, the period between the onset and treatment of the primary lesion and the disclosure of a metastatic focus in the spine can be of different lengths. The mean time between the detection of the primary tumor and the onset of metastasis is 18.9 months. Lung cancer metastases have the shortest period between these events, which is 9 months. The longest period is characterized by metastases in patients with breast or prostate cancer, as they appear in 14.9 and 17.4 months, respectively, from the detection of the primary tumor [3]. The spine is the most frequent site of bone metastasis [2]. In postmortem examinations of neoplastic disease, metastatic foci in the spine are found in 30 to 70% of cases [6]. The primary tumor sites include the breasts, prostate, kidneys, lungs, multiple myeloma, thyroid gland, larynx, ovaries, large intestine, or liver. The frequency of metastases from the locations mentioned above is adequate for the number of people with the corresponding primary tumor in a given population. Their peak incidence occurs in the sixth and seventh decades of life [4,6]. Metastatic tumors of the spine are located in different spine sections (Figure 1). A large group (18%) are multi-site metastases involving more than one section of the spine [6]. Some metastatic neoplasms show a clear predilection to specific parts of the spine, e.g., lung and breast cancers often metastasize to the thoracic section, while cancers of the prostate and colon often metastasize to the lumbar compartment [5,7].

Paget’s “seed and soil” model explains the pathogenesis of spine metastases. It assumes that a seed symbolizes cancer cells and that soil is a specific place where they can multiply. Bone tissue is a site of dynamic metabolic changes, making it a privileged location for developing neoplastic metastases. These constant changes are propelled by the opposing functions of osteoclasts, which lead to bone resorption, and osteoblasts, which promote bone formation. The bone matrix is rich in various growth factors, which can be used as a result of bone turnover by the tumor to multiply faster. It was also noticed that metastases develop well in bones where the trabecular structure predominates and bones containing bone marrow, such as vertebrae and epiphyseal parts of long bones. In the trabecular system in blood vessels, blood perfusion is slower, and the walls are more permeable, promoting neoplastic cells’ retention [8,9].

Considering the effects of the metastatic tumor on the bone, we can divide neoplastic bone metastases into osteoclastic, osteoblastic and mixed lesions. More common are osteoclastic changes, which stimulate osteoclastogenesis. On the other hand, osteoblastic changes promote osteogenesis (Table 1) [5,8,9].

The phenomenon of the formation of metastases in the spine is favored, among others, by its anatomical structure. Cancer cells enter the spine through the venous vessels, but to a lesser extent also via arterial and lymphatic vessels and direct tumor infiltration. The blood drains from the spinal cord, bone, and ligament structures for venous plexuses of Batson. The non-valvular nature of these veins creates specific opportunities for blood in these vessels to flow in both directions. The venous plexuses of Batson are connected with the inferior and superior vena cava system and the portal vein system. These particular conditions of vessel structure and connection with many different venous vessels make the spine particularly susceptible to the spread of neoplastic cells from different parts of the body [2].

Pain is often the first symptom of metastasis and occurs in more than 90% of patients [7,12,13]. It can be divided into root, bone, and mechanical. Root pain is caused by the pressure of the tumor on the roots of the spinal nerves. It is sharp, piercing, and radiates to specified dermatomes. It may disappear with changes in body position. Bone pain results from periosteal stretching, an increase in intraosseous pressure, or the presence of an inflammatory response caused by metastasis. It is characterized by its occurrence in the morning and evening hours. It occurs at the site of the neoplastic lesion and increases with movement. Mechanical pain results from damage to the vertebrae by the spreading neoplastic process in its place, which is a consequence of a violation of the spine’s stability and reduced resistance to loads [14]. The clinical picture is influenced by local factors resulting from damage to the spine, spinal cord, spinal nerve roots, paravertebral structures, and general aspects related to neoplastic disease. The cause of damage to the nerve roots is their compression by fragments of a pathologically fractured vertebra or the neoplastic mass present in the spinal canal and intervertebral foramina. Spine metastases do not have characteristic symptoms, and they are often detected at an advanced stage when they occupy more than one vertebra [3,6,7]. As the disease progresses, patients develop limb paresis, decreased sensation below the level of spinal cord damage, and dysfunction of the bladder and anal sphincters [7,15]. Cancer can also lead to chronic or acute ischemia of the spinal cord [15].

## 2. Scales Assessing the Severity of the Disease, Algorithms Setting Out Treatment Strategies, and Qualifications for the Surgical Treatment

Qualification for surgical treatment and the appropriate comprehensive approach requires an insightful assessment of the clinical patient’s condition. Special attention has to be paid to the stage of advancement of the primary neoplastic disease, already received treatment, general patient’s condition and neurological status, the degree of spinal bone structures damage, and precise location and extent of the metastasis. The combined analysis of these aspects allows for evaluating, whether the patient can be operated on and, if so, how it should be performed [6,16,17]. Surgical treatment focuses on the decompression of the spinal cord and nerve roots and an internal stabilization of the spine. The surgery should alleviate the pain, improve the quality of the patient’s life, and create conditions for the continuation of oncological treatment [18,19]. In the qualification to the surgical treatment, numerous scales are used to evaluate the patient’s condition in general and neurological aspects and define the stage of advancement of the neoplasm disease and damage to the spine. Algorithms and paradigms of conduct are also proposed to facilitate the decision in qualification to the optimal way of treatment.

The Karnofsky scale allows assessing the functional patient’s condition. It determines the degree of patient’s efficiency on a scale from 0 to 100 points, where 100 points define the proper patient’s condition, and gaining 0 points is equivalent to death. The higher the score on this scale, the better the quality of the patient’s life [20].

Before the patient’s qualification for the surgery, the operational risk, according to the guidelines of the American Society of Anesthesiologists (ASA), is assessed [21]. This is a six-point scale. Assignment of the patient to IV-VI grade disqualifies from surgery on spine metastases [22]. 

To assess the patient’s neurological condition, the Frankel scale was used. This scale is five stages (from A to E) [23]. In patients classified as groups A and B, surgical treatment rarely restores the ability to walk. Whereas surgical treatment is often effective for groups C and D and improves the patient’s neurological condition [24]. Nowadays, to assess the spinal cord injuries, the American Spinal Injury Association Impairment Scale (AIS) is used instead the Frankel scale. Evaluation with the Frankel scale can be subjective, whereas AIS allows to more precisely assessment of the neurologic levels of injury. AIS based on an anorectal examination, a dermatomal based sensory examination and a myotomal-based motor examination [25].

The Visual Analogue Scale (VAS) is commonly used to assess the objective intensity of the pain. The patient sets the sensed pain on a 10-point scale, with 0 reflecting lack of pain and 10 marking unbearable pain [26].

Spinal Instability Neoplastic Score (SINS) is a rating system that allows identifying the patients with spine metastases who will benefit from the surgical treatment. This scale takes into consideration six criteria: the localization of the metastasis, the mechanical pain, the kind of the pathological bone lesion, the change of the axis and curvature of the spine, the consequences of the compression fracture of the vertebral body, and the involvement of the posterolateral vertebra elements. If the summed value is between 0 and 6 points, the spine is stable, and there is no indication of surgical treatment unless there are symptoms of spinal cord compression. A result of 7–12 points portends near instability of the spine, and 13–18 points suggest that this instability is present. Obtaining 7 points and more indicates the need for a surgical consultation and indicates the possibility and advisability of the operation [27,28].

De Wald score is used to assess the stage (I–V) of advancement of the neoplastic disease. It takes into account a patient’s immune condition and damage to the spine and nervous elements of the spinal canal. According to this scale, surgical treatment should be considered when more than 50% of the vertebral body is damaged and when both or one of the epiphyses of the vertebral arch is infiltrated by the neoplastic lesion, even if the vertebral body has not collapsed [29].

Tomita’s surgical classification allows the evaluation of the extent of the spine metastases based on the analysis of the magnetic resonance images. It distinguishes seven types of changes (T1–T7) [6]. The types T1–T6 qualify for the total en bloc spondylectomy, but patients with T1–T2 types may be treated by radiotherapy, chemotherapy, corpectomy, or hemivertebrectomy [30].

Based on radiological and neurological examination, the five-stage Harrington scale assesses bone changes in the spine and their consequences [6]. Patients qualified to I, II, and III groups should be treated conservatively. The changes described in IV and V degrees are indications to surgical treatment [24].

Patients with spine metastases often present with spinal cord compression, which degree of intensity is determined by the epidural spinal cord compression scale—ESCC. It distinguishes six degrees of spinal cord compression, where 0 means that the metastasis is limited only to the bone, and 1a is the presence of the metastasis in epidural space, without deformation of the dural sac; 1b points to a deformation of the dural sac, without the spinal cord infiltration; and 1c with its infiltration, but without the compression; 2. and 3. determine the compression of the spinal cord with visible (2) and invisible (3) cerebrospinal liquid surrounding the spinal cord [31]. In degrees from 0 to 1c, with preserved spine stability, radiotherapy may be used as a preliminary treatment. In degrees 2. and 3., if metastasis is not sensitive to radiotherapy, the primary surgical treatment is advocated [32].

According to the Weinstein–Boriani–Biagini classification, a vertebra is divided into 12 zones clockwise and into five layers of localization of the neoplastic tissue (A–E). It allows the evaluation of which sector of the vertebra the metastasis is present and gives the opportunity of the surgical approach [33].

The Asdourian scale is based on magnetic resonance examination and assesses the localization of the metastasis and the spine’s stability. The changes are marked with B in a cervical and lumbar segment, and with A in the thoracic segment. Types IA and IB do not cause instability of the spine and can be treated conservatively. In types IIA, IIB, IIIA, and IIIB, axial instability is present, especially with the destruction of the back of the vertebrae. These cases qualify for surgical treatment. Type IVA is characterized by progressive displacement instability and deformation of the spine. The treatment is surgical, involving the decompression of the spinal cord and nerve roots [6,34].

One of the most widely used classifications is the Tokuhashi scale. It was developed to evaluate the prognosis and survival time of the patient with tumor metastases to the spine and choose an appropriate treatment. The assessment of the patient takes into account the general condition (according to the Karnofsky Performance Scale (KPS)), amount of metastatic foci apart from the spine, amount of spine metastases, metastases to the internal organs, primary location of the tumor, and a degree of the spinal cord damage. This scale is often used because it is not influenced by any therapeutic factor [35]. Based on this scale, the patients are divided into three groups: 0–8, 9–11, and 12–15 points with predicted survival times less than 6, ≥6, and ≥12 months, respectively. In the first group, only conservative treatment or palliative surgery is recommended. Patients within the point range of 9–11 receive palliative surgery or, rarely, excisional procedures, whereas the highest-point group is assigned for radical surgical treatment [35,36].

The modified Bauer scale also allows for predicting the patient’s survival time. It is based on the etiology of a primary tumor and the absence or presence of visceral metastases, lung cancer, and one solitary skeletal metastasis. It divides the patients into three groups: with a median survival time of 4.8 months (no indications for surgical treatment), 18.2 months (surgical palliative treatment by posterior approach), and 28.4 months (surgical treatment) [35].

The Tomita scale allows the assignment of the patient to an appropriate treatment based on three factors: a primary tumor grade, metastases to the internal organs (lungs, brain, liver, and kidneys), and metastases to the bones. The individual factors have explicitly been underlined. Lower point values qualify for a resection of the neoplastic lesion with an oncological margin and higher for palliative surgical treatment or supportive treatment [37]. Table 2 is a summary of the discussed scales (Table 2).

Treatment algorithms are widely based on the concept of above-mentioned classifications. One of them, NOMS, proposes the choice of treatment on the basis of neurological (myelopathy and grade of epidural spinal cord compression, ESCC), oncological (tumor sensitivity to radiation), mechanical (spine stability), and systemic (the patient’s general condition, ability to tolerate the procedure) aspects [38,39]. Another paradigm, proposed by Gasbarrini A. et al., focuses on the assessment of the patient according to ASA scale and on determining the patient’s neurological condition and the sensitivity of the tumor to adjuvant, non-surgical treatment. This assessment allows a decision to be made about the direction of the therapy [22].

Unfortunately, none of the scales and paradigms is perfect. Some of the presented scales were created many years ago, and now they are not necessarily adequate. Each patient is different in medical history, physical condition, and psychosocial circumstances. Therefore, the therapeutic approach should be individualized in each case. 

## 3. Diagnostics

### 3.1. Imaging

#### 3.1.1. X-ray Examination

Plain radiographs can present the damage of the vertebral column: such as wedge compression, fracture dislocation, the collapse of the vertebrae, as well as paraspinal soft-tissue shadows. Changes are visible on radiograms only when the bone is destroyed in 50–75% [12,40,41]. The pathognomonic ‘winking owl sign’ is visible in the p-a projection and signifies damage [42]. Sometimes metastases form small cavities, ca. 1 mm in diameter, which make the bone less visible on a plain film. The blurred outline of the vertebrae’s pedicle is indicative of an infiltration of the cortical bone. It is a distinctive sign of a neoplasm, which should be looked out early on in the diagnostic process. There are few indications that the metastasis might be malignant: one-sided damage, irregular distortion of the articular surface on vertebrae’s pedicle, involvement of the soft tissue around the spine, and destruction of the base of the arch. The upper thoracic location is also significant for high malignancy. A radiogram is an easily accessible examination, and it is frequently used for monitoring metastases. Due to its ability to show cortical bone, it is useful for fracture risk assessment [43].

#### 3.1.2. Magnetic Resonance Imaging (MRI)

MRI is the examination of choice in case of metastatic changes in the spine [43]. The primary components of bone marrow are water and fat, and MRI shows the contrast between them. With age, the amount of each constituent shifts, e.g., percentage of fat increases. At birth, it equals 40%, and when red bone marrow is replaced with yellow marrow, the amount of fat rises to 80%. Therefore, it is essential to distinguish physiological changes from neoplastic ones.

The proliferation of cells in osteoblastic metastases results in an increased quantity of water, which attenuates the signal in T1w images which becomes either isointense or hypointense in comparison to surrounding muscle tissue or disc. In cases like this, changes often have stronger signals in STIR images. In osteoclastic metastases, it is the other way round—the decreased amount of cells results in a higher percentage of fat, which intensifies the signal in T1w images. It looks similar to osteoporosis or post-radiotherapy changes. Displayed pathologies enhance after administration of paramagnetic contrast [44]. A halo sign, a bright rim around the osteoblastic tumor, may appear in T2w and STIR sequences. It indicates the presence of fluid in the bone [45]. MRI visualizes the presence of neoplasm in the spinal canal (in epidural space), extent of spinal cord compression, hemorrhagic and ischemic changes in the spinal cord, as well as necrosis [43].

Intravenous administration of gadolinium may significantly improve visibility of metastasis in T1-weighted image, especially when it is located outside the spinal canal, but in the case of osteoblastic neoplasms, improvement is slight, if at all. To better visualize metastases on T2w images, USPIO (ultra-small superparamagnetic iron oxide) nanoparticles can be administered. They attenuate the signal of normal bone marrow, thus making the metastasis apparent as it stays hyperintense. After radiation treatment, MRI is used for differentiation between post-radiation changes and the recurrence of the neoplasm [43].

#### 3.1.3. Computed Tomography (CT)

The resolution of images in CT scans is around ten times higher than in plain films. Spine CT shows the extent of bone destruction, particularly in trabecular bone, and is more sensitive and specific than X-ray because of its higher resolution. Osteolytic lesions have a more distinct outline on CT scans than on plain films. Moreover, CT enables visualization of necrosis, calcifications, destruction of the pedicle and cortical bone, epidural lesions, and expansion of neoplasm to paraspinal tissues. In osteoblastic metastases, bone trabeculae are less visible, and the border between the cortical and trabecular bone is blurred. Detailed imaging of the pedicles in CT can help with acquiring the proper trajectory during the vertebroplasty (posterolateral or through the pedicle) [43].

#### 3.1.4. Bone Scintigraphy

Technetium (99mTc) has a strong affinity to bone and concentrates in places of increased metabolism and strong blood supply. In scintigraphic examination the chosen tracer shows the place of the increased bone turnover [40,45]. Osteoblastic changes, described as ‘hot’ are characterized with increased accumulation of the tag, and osteolytic changes, described as ‘cold’ have decreased accumulation of the tag. The hot lesions are the more common; however, cold changes may indicate that the aggressiveness of the neoplasm [46].

### 3.2. Invasive Diagnostics—Biopsy

Confirming pathology of the tissue is required before implementation of the appropriate treatment [47]. Except for that, the biopsy can identify tissue markers and influence the choice of specific treatment [48].

Depending on anatomical features and supplementary imaging of CT or fluoroscopy, access to the lesion is gained through the pedicle or outside of it. The risk of complications and the accuracy of the biopsy grow with the gauge of the needle. Accuracy of spine biopsy reaches 80%, and it is higher in the case of osteolytic rather than osteoblastic metastases, and it is also higher in the case of metastases rather than primary neoplasms [48].

The percutaneous biopsy is an elegant alternative to open biopsy [47]. Thin needle biopsy (with needle 22–25) or core needle biopsy (with needle 11–15) are performed under control of X-ray, CT, or ultrasonography with local anesthesia [49,50]. Ideally, the histopathologist is present during the procedure and directly evaluates the obtained material under microscopy, which guarantees the utility of the sample for further diagnostics. Such practice—Rapid On Site Evaluation (ROSE)—increases the chances for the diagnostic value of biopsy by >12%. Thin needle biopsy is performed when the tumor is located in paraspinal areas, and core needle biopsy is executed when the bone is affected by neoplasm. The sensitivity of the biopsy is defined in 93.3–96% [49].

Transpedicular biopsy enables a relatively straightforward way to most lesions and is relatively safe—it omits nerves, vessels, lung, and spinal cord. Only in selected cases, the posterolateral or transcostovertebral approach in thoracic is used. However, it carries a serious risk of pulmonary complications, e.g., pneumonia and pneumothorax. Complications of percutaneous biopsy occur in up to 26% of cases and include paraspinal hematoma, haemothorax, damage to the nerve roots, temporary paresis, paraplegia, meningitis, and in very rare cases, death [47].

## 4. The Ways of the Surgical Treatment

Obtaining a good result from surgical treatment enables the continuation of radiotherapy, chemotherapy, hormone therapy, and targeted molecular therapy [17,18,19,51].

Surgical treatment should meet three basic criteria. The first is to provide optimal conditions for restoring the function of the spinal cord and the spinal roots by decompression. The next one is to restore the correct anatomical relations of the spine by adjusting the displacement, restoring the height of the spinal body, and the physiological curves of the spine. The last stage of the surgery is to ensure the internal stabilization of the spine [18,19,51,52,53].

In recent years, progress in surgical treatment has been achieved thanks not only to the development of modern neuroimaging techniques but also to neuroanesthesiology and the implementation of various approaches and surgical methods. Circular relief from compression of the spinal cord is desirable. Different accesses to the spine are used depending on the location of the lesions: anterior approach, posterior approach, anterolateral, posterolateral, and combined multi-stage surgical approaches (Table 3) [6,18,19,52,54,55].

After resection of the tumor and release from compression of the spinal cord and roots of the spinal nerves, it is necessary to ensure anastomosis and an internal stabilization of the spine. Anastomosis and an internal stabilization of the spine are usually repair procedures aimed at restoring the durability of the spine. However, prophylactic stabilization is also carried out when the ongoing disease process may threaten the stability of the osteoarticular-ligamentous system, e.g., the risk of pathological fracture [6,17,19,51,52,55].

The site of the removed tumor in the vertebral body may be filled with bone cement, or the removed backbone is replaced by a prosthesis of the vertebral body (in the shape of a cylinder, a basket filled with plastic (palakost, acrylic cement) [16,71,72,73,74]. A stabilizer using appropriate screws or hooks is attached to the undamaged and durable sites in the spine, above and below the lesion, thus preventing the collapse of the vertebrae, thereby protecting the spinal cord and nerve root against damage. The effectiveness of the stabilization carried out from the front is determined by the lack of damage to the rear column, and the shafts in which the bolts are mounted must be strong enough to carry loads. In the case of the destruction of structures in the posterior spinal column, it is necessary to perform transpedicular fixation involving the introduction of vertebral arches through the base mounting bolts, which are screwed rods. It is the most widely used implant system in surgery for spine metastases. The good results of this type of stabilization were reported by Boucher in 1959 [17,19,55,71,73,75].

Steel implants have a tolerance to rusting because of the special surface. The surface of implants is covered by a layer of non-corroding metals. Mechanical or chemical damage to the protective layer brings corrosion. Bone tissue, nervous tissue, paraspinal tissue in close proximity to the prosthesis is affected by corrosion products. The substances are losing to surrounding tissues and are triggering unprofitable metabolic reactions, inflammatory responses, immune reactions, and oncogenic reactions [76,77].

Providing the internal stabilization of the spine enables the patient to be mobilized very early, without external immobilization of the spine (orthosis) [19,51,55,71]. The metal elements used for stabilization do not preclude the use of subsequent radiotherapy after the surgery [51].

The titanic implants trigger artifacts that handicap postoperative imaging by CT and MRI. CT and MRI are necessary to appraise the radical nature of the operation and allow the project of stereotactic radiotherapy (precise placement and dose of radiation). Both conventional radiotherapy and stereotactic radiotherapy are constricted because the implant consumes radiation. Nowadays, implants with low absorption of radiation are preferred. These implants reduce the number of artifacts. Currently, the PEEK implants (polyetheretherketone) and carbon implants are available [78].

Surgical removal of spine metastases, particularly renal cell carcinoma, thyroid, breast, melanoma, and hepatocellular carcinoma, carries the risk of significant blood loss. To reduce blood loss during surgery and shorten its duration, preoperative embolization is performed. Embolization involves selective occlusion of tumor vessels by permanent or less, absorbable materials embolization [17,51,53,79,80,81,82].

Despite effective embolization, blood loss during surgery can be significant—up to 2 l and more [17,53]. Factors such as the morphology of the tumor, its size, the infiltration of tissues may affect the final blood loss during surgery. There is little evidence in favor of preoperative embolization of spinal metastases [17,53,79,80,81]. The pain relief after embolization is reported in the literature, but that effect is transient [79]. Embolization is considered a rather safe procedure. The possible complications include neurological deficits resulting from blocking the blood supply to the anterior or posterior spinal artery, which may cause spinal cord infarction [79,80,82].

In addition to massive intraoperative bleeding, other complications may occur during tumor removal. They appear sporadically and concern damage to the dura mater, anatomical structures located in the paraspinal area: aorta, main veins—upper/lower, odd veins, lymphatic trunk, esophagus, parotid gland, pleura, peritoneum, nerves, including the recurrent larynx, and branches of the nerve plexuses [17,52,54,56,83]. The consequence of damage to the dura mater may be a difficult-to-heal cerebrospinal fluid leakage from a postoperative wound [18,83]. Other complications may involve the formation of hematoma at the operations site, or wound infection [52,56,83,84]. Complications related to the internal stabilization of the spine include improper placement of the implant, displacement of implants related to loosening of screws screwed into the bone, or breakage of transpedicular screws [52,54,55]. Moreover, thromboembolic complications, circulatory failure, pneumonia, inflammation of the urinary system, and bleeding from the gastrointestinal tract and bladder are also observed. Radiotherapy and chemotherapy administered before surgery increase the incidence of postoperative complications [18,19,80,85,86]. With the proper selection of patients for surgery, the complication rate should not exceed 5%. However, the analysis of the literature is often given a higher complication rate [6].

Minimally invasive surgery (MIS) is used to treat tumor spine metastases in selected cases. MIS is aimed at decreasing postoperative trauma, reducing blood loss, and reducing postoperative complications. Moreover, the patient’s stay in the hospital is shortened. The times to begin oncological treatment—radiotherapy and chemotherapy are shorter [87]. MIS comprises endoscopic video-assisted thoracoscopic surgery (VATS), performed in the thoracic spine. Another way used in minimally invasive surgery is minimal access spine surgery (MASS). MASS uses a surgical microscope or endoscope. In addition, transcutaneous intromission transpedicular screws are used in MIS [88,89].

Another minimally invasive surgical intervention is vertebroplasty performed under regional anesthesia with X-ray control. The bone cement (polymethylmethacrylate, PMMA) is given transcutaneously with the needle to the body of a vertebra attacked by a tumor add to the tumor mass preferably. It results in augmentation of the destroyed vertebral body, improved stabilization of the spine, and decreased pain. Pain is the main indication for vertebroplasty. The procedure should not be done on asymptomatic patients and in the case of spinal cord compression. Kyphoplasty is the procedure’s modification used to regain the height of the broken vertebra and reduce the risk of dislocation of the bone cement to the spinal canal. The balloon is first introduced into the vertebral body and makes the space for bone cement [90,91].

## 5. Radiotherapy

Radiotherapy is one of the main methods of treating patients with metastatic neoplasms in the spine. Conventional external beam radiotherapy (EBRT) is the most commonly used form of treatment. Its effectiveness and the size of the dose used are closely related to the sensitivity of the tumor to radiation, which is related to its histological structure [92]. Radiation-sensitive tumors include those of hematological origin, such as lymphoma or multiple myeloma, and some solid tumors, including breast or prostate cancer. Most solid tumors, such as thyroid cancer, liver cancer, or melanoma, are resistant to radiotherapy [38]. Standard radiation doses for this method are 8 Gy in one fraction, 20 Gy in 5 fractions, or the most popular: 30 Gy in 10 fractions. The limitation here is the potential damage to the spinal cord because conventional radiation therapy also significantly impacts anatomical structures in the tumor area [92].

Innovative radiotherapy methods, such as stereotactic radiosurgery (SRS) and stereotactic body radiotherapy (SBRT), are a promising alternative. It allows the use of up to 3 times higher doses than in the case of EBRT, which is concentrated precisely at the tumor site. It is possible thanks to the precise stabilization of the patient and modulation of the intensity of the radiation. SRS is usually used as a single dose of radiation, while SBRT is used in two to five doses [92]. The increased amount of radiation enables the treatment of neoplasms that are radio-resistant and insensitive to conventional radiotherapies, such as melanoma. This is because apart from destroying the double-stranded DNA of cancer cells, the vascular network inside the tumor is also destroyed. These methods ensure a high local tumor control rate, regardless of its histological origin. It is also beneficial from the patient’s point of view that there are fewer doses than in the case of EBRT [38], but the determination of the dose to be used is a subject of discussion [92]. The most severe complication of radiotherapy is myelopathy, with a risk of approximately 0.5%. Other complications of SRS include dysphagia, diarrhea, mucositis, and compensatory vertebral fractures, with a risk of up to 40%. In comparison with conventional radiotherapy, the risk is only 5% [38]. It should be mentioned here that the administration of bisphosphonates prevents pathological vertebral fractures.

## 6. Other Ways of Treatment

Apart from preventing pathological vertebral fractures, Bisphosphonates also show a protective effect against bone metastases and increase bone mass density in the lumbar region by 3–4%. Moreover, they prevent the onset of osteoporosis in women [93]. An example of a bisphosphonate is zoledronic acid (ZA), which is used primarily to prevent spinal cord compression resulting from bone tissue wasting caused by tumor metastasis to the spine. It is optimally administered intravenously at a dose of 4 mg every 4 weeks. In these cases, Denosumab, a human monoclonal antibody that strongly binds the receptor activator for Nuclear Factor κ B Ligand (RANKL), has comparable effectiveness to zoledronic acid (ZA), thus inhibiting osteoclast maturation and bone tissue resorption, which is great importance in the prevention of compression of the spinal cord, bone tissue destroyed by cancer. It is subcutaneously administered at a dose of 120 mg every 4 weeks [94].

In the case of spinal cord compression by metastasis, corticosteroids are the first treatment line. They should be given immediately. Their exact mechanism of action and dosing are a matter of debate, but they reduce angioedema and relieve inflammation in the area of metastasis. There are no precise guidelines for the dosage of corticosteroids, and they are selected individually to case [92]. Side effects of their use include, among others: exacerbation of diabetes, decreased immunity, hypertension, and peptic ulcers [12]. The use of dexamethasone is particularly recommended to treat metastatic prostate cancer in the spine. In addition, to improving treatment outcomes, it also alleviates the pain experienced by the patient as a result of the metastasis that has arisen [95]. Methylprednisolone is used to treat follicular thyroid cancer (FTC), a frequent complication of which is metastasis to the spine. In the presence of a sodium-iodide symporter (NIS-sodium-iodide symporter), radioiodine therapy (RAI) is the first-line treatment. It uses the iodine ^131^ I isotope. Additionally, in the treatment of FTC, TSH suppression therapy is used to inhibit the impulses to further tumor growth [96].

Chemotherapy is mainly used as adjuvant therapy. Its effectiveness depends on the histological type of the primary tumor. Tumors sensitive to this type of treatment include lymphoma, seminoma, and neuroblastoma [92]. In the treatment of breast cancer, which is the most common source of metastases to the spine, the use of vinorelbine or taxanes may prove effective. However, in the light of the latest knowledge, aromatase inhibitors, which include letrozole, exemestane, anastrozole, and fulvestrant, seem to be the right choice [97]. Another cancer that frequently metastasizes to the spine is prostate cancer. In this case, docetaxel is the first-line treatment as add-on therapy. Cabazitaxel is used as the second-line treatment. However, it has more side effects, including anemia and leukopenia [98].

## 7. Therapeutic Perspectives

A good therapeutic effect in the fight against metastases to the spine (breast cancer with overexpression of the HER2 receptor, clear cell carcinoma of the kidney, colorectal cancer, lung cancer, melanoma) is achieved by the use of ionizing radiation in combination with molecular targeted therapy, including inhibitors of systemic checkpoints immune. In such a case, it is believed that better results are achieved by using radiotherapy earlier in several fractions than in one [38]. Examples of immune checkpoint inhibitors are ipilimumab, which binds the CTLA-4 antigen of T cells, resulting in an increase in their activity, and nivolumab, which binds to the PD-1 programmed cell death receptor, which blocks its function. These agents increase the number of T lymphocytes capable of responding against the tumor, which, after prior radiotherapy, brings satisfactory results. In addition, vascular endothelial growth factor (VEGF) inhibitors, in combination with SRS, as exemplified by Axitinib, can be used to combat spinal metastasis. They lead to endothelial dysfunction by inhibiting the acid sphingomyelinase pathway [38].

## 8. Prognosis

The primary role of surgical interventions in spine metastases is focused on further damage prevention, not radicality of tumor excision [17,18,19].

The average survival is 6 months, with the longest period of over a year for metastasis from the prostate, thyroid, rectum, and colon and the shortest for the stomach, which counts less than 3 months [4,6]. The median survival in patients with breast cancer and bone metastases is 16 months, but with the coexistence of various complications, only 7 months. Patients with lung cancer and bone metastases have a worse prognosis, and the estimated survival time is 6–12 months. Moreover, the coexistence of renal cancer and bone metastasis is also associated with a short survival time [84,99].

After surgery, in up to 90% of patients, significant pain reduction and improved overall physical and neurological functioning are observed. Those increase the quality of life but do not directly influence survival time [18,51,71].

Surgical intervention has better results than radiotherapy only [100]. It is estimated that in 50–80% of patients undergoing radiotherapy, pain associated with metastases was relieved, about one-third completely [101]. Radiotherapy can be used as monotherapy. However, the best results may be obtained when it is carried out after the surgery [5,38].

It has also been proven that bisphosphonates and denosumab, as an extension to conventional treatments, may reduce mortality from bone metastasis [99].

The patient’s condition and the nature of the tumor have a tremendous impact on the survival time. One of the most important prognostic factors is the patient’s neurological function [51,71,100,102]. An unfavorable prognosis concerns patients who show rapid progression of symptoms of spinal cord injury [57]. 

Patients in whom metastasis to the spine and its ailments is the first symptom of cancer (unknown primary focus) are less likely to achieve a good treatment outcome [84].

The site of metastasis in the spine may be an essential factor influencing the prognosis [103,104]. 

Tumor metastases limited to the bone only have a better prognosis than metastases located in the visceral organs [19]. Pathological fractures caused by spine metastases concern about 10% of all patients and significantly increase mortality in this group (annual survival rate is estimated around 22–40%) [84].

Treatment is often initiated in patients with metastases to the spine in the advanced stage of cancer when the primary tumor is large (over 2 cm); hence, the prognosis is poor [102].

Guzik pointed out that the worst results in terms of neurological evaluation after surgery were obtained in patients with lung and kidney cancer and, best of all, with breast cancer and myeloma [71].

Advances in diagnosis, treatment, and care of patients with spine metastases improve prognosis in the quality of life of those patients. However, they do not significantly prolong the survival time [5,19,84]. It is not only a generalized neoplastic disease that leads to death but also complications related to spinal cord damage, such as multi-organ failure, deep vein thrombosis of the lower limbs, thromboembolism, intestinal obstruction, pneumonia, urinary tract infection, or pressure ulcers [103].

## 9. Conclusions

**Figure 2:**
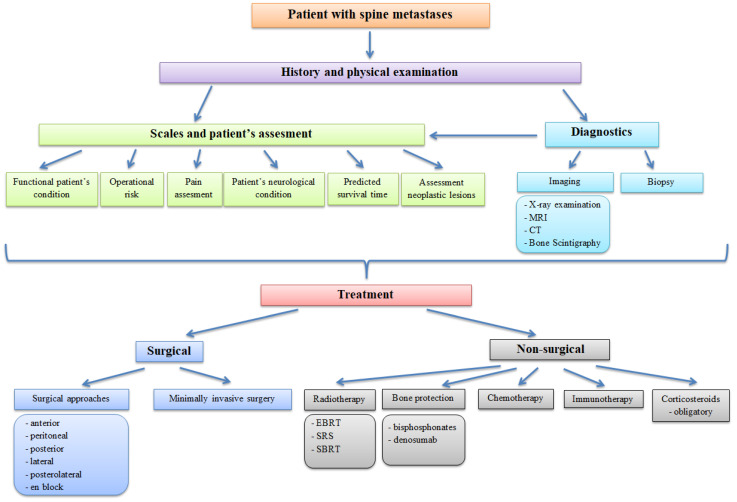
The algorithm summarizing the diagnostic and treatment strategy for patients with spinal metastases, based on data presented in the review.

Spine metastases result from the distant and adjacent spread from primary lesions of many neoplasms [6]. In diagnostics of the metastases, MRI, CT, and bone scintigraphy are often used [43]. The qualification for the right way of treatment requires multifaceted patient assessment performed by many specialists. Scales, algorithms, and paradigms are beneficial. They allow evaluation of the patient’s condition, the stage of advancement of the neoplasm disease, and the prognosis [5]. Surgical treatment of the spine metastases is palliative. Its main focus is relieving the pain, preventing neurological deficits from arising, restoring spine stability, and, more importantly, inhibition of disease progression [19,71]. Minimally invasive surgery reduces postoperative complications and enables faster implementation of oncological treatment [87]. In the radiotherapy of the spine metastases, the SRS is gaining popularity, which allows using higher doses of radiation than in ERBT, concentrated precisely at the tumor site. Using corticosteroids during therapy, which decrease angioedema in the area of the metastasis, alleviate pain and improve treatment results, is important [92]. Immunotherapy is also gaining more popularity, improving prognosis even in advanced metastatic disease [38]. The oncological disease is shifting more and more from being lethal to rather chronic. The overall survival of oncological patients is increasing. Therefore, the probability of developing metastases, also to the spine, rises. However, they can usually be controlled for many years with systemic therapies. That leads to a paradigm shift from being therapeutically radical to the good control of the disease, maintaining the satisfactory quality of life and complications prevention. For this reason, the holistic approach to the disease, which means choosing the best therapeutic option for the patient and ensuring appropriate mental and emotional support; hence the importance of the therapeutic team in the treatment process, is critical (Figure 2) [19].

## Figures and Tables

**Table 1:** Type of spine metastases in terms of its effect on bones.

Type of Spine Metastases		
	Osteoclastic	Osteoblastic
**Primary tumor**	e.g., lungs, kidneys, multiple myeloma	e.g., prostate
**Factors secreted by neoplasm cells**	IL-1 IL-6 IL-8 IL-10 IL-11 TGF-α TGF-β PTHrP PgE2 TNF CSF-1 GM-CSF M-CSF VEGF EGF	ET-1 TGF-β PDGF BMP IGF FGF CXCL-1 uPA PSA
**Factors released from the bone microenvironment**	TGF-β	
	FGF	
	IGF	
	BMP	
	PDGF	
	OPG	
	HGF	
	IL-6	
	CTGF	
	M-CSF	
	VEGF	
	PTHrP activins	

The factors released by tumor cells and bone microenvironment: interleukin-1 (IL-1), interleukin-6 (IL-6), interleukin-8 (IL-8), interleukin-10 (IL-10), interleukin-11 (IL-11),transforming growth factor α (TGF-α), transforming growth factor β (TGF-β), parathyroid-related peptide (PTHrP), prostaglandin E2 (PgE2), tumor necrosis factor (TNF), colony stimulating factor (CSF-1), granulocyte macrophage-colony stimulating factor (GM-CSF), monocyte-colony stimulating factor (M-CSF), vascular endothelial growth factor (VEGF), epidermal growth factor (EGF), endothelin-1 (ET-1), platelet-derived growth factor (PDGF), bone morphogenetic protein (BMP), insulin-like growth factor (IGF), fibroblast growth factor (FGF), C-X-C motif chemokine ligand 1 (CXCL-1), urokinase-tipe plasminogen activator (uPA), prostate specific antigen (PSA), osteoprotegerin (OPG), hepatocyte growth factor (HGF), connective tissue growth factor (CTGF) [2,5,8,9,10,11].

**Table 2:** The discusses scales and their application [6,20,21,23,24,25,26,27,28,29,30,31,32,33,34,35,36,37].

Scales Assessing the Severity of the Disease	
The Name of the Scale	What Does the Scale Evaluate?
The Karnofsky scale	functional patient’s condition
ASA Physical Status Classification System	operational risk
the Frankel scale, the American Spinal Injury Association Impairment Scale (AIS)	patient’s neurological condition
The Visual Analogue Scale (VAS)	pain assessment
Spinal Instability Neoplastic Score (SINS), De Wald scale, Tomita’s surgical classification, Harrington scale, epidural spinal cord compression scale (ESCC), Weinstain–Boriani–Biagini classification, Asdourian scale, Tomita scale, Tokuhashi scale	assessment neoplastic lesions, their advancement and localization in bone and ligamentous structures of the spine and the spinal canal
Tokuhashi scale, modified Bauer scale	predicted survival time of a patient with neoplastic metastases to the spine

**Table 3:** Surgical approaches [17,56,57,58,59,60,61,62,63,64,65,66,67,68,69,70].

Approach	Level of Spine	Description
anterior	the upper part of the cervical spine, craniospinal junction: the base of the skull, atlas (C1), and axis (C2)	through the posterior wall of the pharynx, mandibular (with or without cut of the mandible)
	C3 to C7	proposed by Clovard Smith and Robinson—in front of the anterior edge the sternocleidomastoid muscle
	thoracic vertebrae	the anterior approach to the thoracic vertebrae is complex from Th2 to Th5 due to the limitations of the sternum (the need for sternotomy) and to the Th10 to L1 thoracolumbar spine junction caused by diaphragm attachments (the need to connect the post-pleural and retroperitoneal entrances)
	lumbar shafts L1 to L4	provided by retroperitoneal access; the peritoneal approach enables surgery in the L5 vertebrae and sacral segments
peritoneal	L5 sacral segments	-
posterior	-	routinely used laminectomy, which makes way to open the vertebral canal and exposes posterior surface of spinal cord
lateral	-	laminectomy and total excision of pedicles of vertebral arch at the same time enable lateral approach to vertebral canal and show lateral surface of spinal cord (intrapedicular approach); the excision of the intervertebral joint and head of rib in thoracic spine expose lateral side of vertebral body (lateral approach—costotransversectomy and postpleural lateral approach)
posterolateral	-	the body of the thoracic vertebra can be visualized via a posterolateral thoracotomy
en block	-	tumor excision in one piece with margin of surrounding tissue; the character of metastatic tumor and localization in the spine rarely allows the use of this kind of resection; usually debulking is used, the aim is to remove the pressure of the spinal cord

## References

[1] Chi J.H., Bydon A., Hsieh P., Witham T., Wolinsky J.P., Gokaslan Z.L. (2008). Epidemiology and demographics for primary vertebral tumors. Neurosurg. Clin. N. Am..

[2] Maccauro G., Spinelli M.S., Mauro S., Perisano C., Graci C., Rosa M.A. (2011). Physiopathology of spine metastasis. Int. J. Surg. Oncol..

[3] Hong S., Youk T., Lee S.J., Kim K.M., Vajdic C.M. (2020). Bone metastasis and skeletal-related events in patients with solid cancer: A Korean nationwide health insurance database study. PLoS ONE.

[4] Phanphaisarn A., Patumanond J., Settakorn J., Chaiyawat P., Klangjorhor J., Pruksakorn D. (2016). Prevalence and Survival Patterns of Patients with Bone Metastasis from Common Cancers in Thailand. Asian Pac. J. Cancer Prev..

[5] Lorkowski J., Grzegorowska O., Kozień M.S., Kotela I. (2018). Effects of Breast and Prostate Cancer Metastases on Lumbar Spine Biomechanics: Rapid In Silico Evaluation. Adv. Exp. Med. Biol..

[6] Guzik G. (2017). Current Incidence of Different Morphological Types of Malignant Metastases to the Spine Based on Magnetic Resonance Imaging. Ortop. Traumatol. Rehabil..

[7] Gilbert R.W., Kim J.H., Posner J.B. (1978). Epidural spinal cord compression from metastatic tumor: Diagnosis and treatment. Ann. Neurol..

[8] Fornetti J., Welm A.L., Stewart S.A. (2018). Understanding the Bone in Cancer Metastasis. J. Bone Miner. Res..

[9] Yin J.J., Pollock C.B., Kelly K. (2005). Mechanisms of cancer metastasis to the bone. Cell Res..

[10] Ritchie C.K., Andrews L.R., Thomas K.G., Tindall D.J., Fitzpatrick L.A. (1997). The effects of growth factors associated with osteoblasts on prostate carcinoma proliferation and chemotaxis: Implications for the development of metastatic disease. Endocrinology.

[11] Lin S.C., Yu-Lee L.Y., Lin S.H. (2018). Osteoblastic Factors in Prostate Cancer Bone Metastasis. Curr. Osteoporos. Rep..

[12] Jacobs W.B., Perrin R.G. (2001). Evaluation and treatment of spinal metastases: An overview. Neurosurg. Focus.

[13] Ecker R.D., Endo T., Wetjen N.M., Krauss W.E. (2005). Diagnosis and treatment of vertebral column metastases. Mayo Clin. Proc..

[14] Vialle L.R., Gokaslan Z.L., Boriani S., Fisher C.G. (2015). Metastatic Spinal Tumors.

[15] Yasui H., Ozawa N., Mikami S., Shimizu K., Hatta T., Makino N., Fukushima M., Baba S., Makino Y. (2017). Spinal Cord Ischemia Secondary to Epidural Metastasis from Small Cell Lung Carcinoma. Am. J. Case Rep..

[16] Eleraky M., Setzer M., Vrionis F.D. (2010). Posterior transpedicular corpectomy for malignant cervical spine tumors. Eur. Spine J..

[17] Clausen C. (2017). Preoperative embolization in surgical treatment of metastatic spinal cord compression. Dan. Med. J..

[18] Boussios S., Cooke D., Hayward C., Kanellos F.S., Tsiouris A.K., Chatziantoniou A.A., Zakynthinakis-Kyriakou N., Karathanasi A. (2018). Metastatic Spinal Cord Compression: Unraveling the Diagnostic and Therapeutic Challenges. Anticancer. Res..

[19] Curtin M., Piggott R.P., Murphy E.P., Munigangaiah S., Baker J.F., McCabe J.P., Devitt A. (2017). Spinal Metastatic Disease: A Review of the Role of the Multidisciplinary Team. Orthop. Surg..

[20] Péus D., Newcomb N., Hofer S. (2013). Appraisal of the Karnofsky Performance Status and proposal of a simple algorithmic system for its evaluation. BMC Med. Inform. Decis. Mak..

[21] ASA Physical Status Classification System Committee of Oversight: Economics, guidelines of the American Society of Anesthesiologists. 2014, Revision from 2020. https://www.asahq.org/standards-and-guidelines/asa-physical-status-classification-system.

[22] Gasbarrini A., Cappuccio M., Mirabile L., Bandiera S., Terzi S., Barbanti Bròdano G., Boriani S. (2004). Spinal metastases: Treatment evaluation algorithm. Eur. Rev. Med. Pharmacol. Sci..

[23] Frankel H.L., Hancock D.O., Hyslop G., Melzak J., Michaelis L.S., Ungar G.H., Vernon J.D., Walsh J.J. (1969). The value of postural reduction in the initial management of closed injuries of the spine with paraplegia and tetraplegia. Spinal Cord.

[24] Mazurkiewicz T. (2006). Surgical management of bone metastases of the spine. Ortho Trauma.

[25] Roberts T.T., Leonard G.R., Cepela D.J. (2017). Classifications in Brief: American Spinal Injury Association (ASIA) Impairment Scale. Clin. Orthop. Relat. Res..

[26] Delgado D.A., Lambert B.S., Boutris N., McCulloch P.C., Robbins A.B., Moreno M.R., Harris J.D. (2018). Validation of Digital Visual Analog Scale Pain Scoring with a Traditional Paper-based Visual Analog Scale in Adults. J. Am. Acad. Orthop. Surg. Glob. Res. Rev..

[27] Fox S., Spiess M., Hnenny L., Fourney D.R. (2017). Spinal Instability Neoplastic Score (SINS): Reliability Among Spine Fellows and Resident Physicians in Orthopedic Surgery and Neurosurgery. Glob. Spine J..

[28] Fisher C.G., DiPaola C.P., Ryken T.C., Bilsky M.H., Shaffrey C.I., Berven S.H., Harrop J.S., Fehlings M.G., Boriani S., Chou D. (2010). A novel classification system for spinal instability in neoplastic disease: An evidence-based approach and expert consensus from the Spine Oncology Study Group. Spine.

[29] DeWald R.L., Bridwell K.H., Prodromas C., Rodts M.F. (1985). Reconstructive spinal surgery as palliation for metastatic malignancies of the spine. Spine.

[30] Tomita K., Kawahara N., Murakami H., Demura S. (2006). Total en bloc spondylectomy for spinal tumors: Improvement of the technique and its associated basic background. J. Orthop. Sci..

[31] Bilsky M.H., Laufer I., Fourney D.R., Groff M., Schmidt M.H., Varga P.P., Vrionis F.D., Yamada Y., Gerszten P.C., Kuklo T.R. (2010). Reliability analysis of the epidural spinal cord compression scale. J. Neurosurg. Spine.

[32] Quraishi N.A., Arealis G., Salem K.M., Purushothamdas S., Edwards K.L., Boszczyk B.M. (2015). The surgical management of metastatic spinal tumors based on an Epidural Spinal Cord Compression (ESCC) scale. Spine J..

[33] Boriani S., Weinstein J.N., Biagini R. (1997). Primary bone tumors of the spine. Terminology and surgical staging. Spine.

[34] Patt J.C., Leas D.P., Marco R.A.W. (2018). Spinal Instability in Metastatic Disease. Metastatic Spine Disease.

[35] Tokuhashi Y., Uei H., Oshima M., Ajiro Y. (2014). Scoring system for prediction of metastatic spine tumor prognosis. World J. Orthop..

[36] Tokuhashi Y., Matsuzaki H., Oda H., Oshima M., Ryu J. (2005). A revised scoring system for preoperative evaluation of metastatic spine tumor prognosis. Spine.

[37] Tomita K., Kawahara N., Kobayashi T., Yoshida A., Murakami H., Akamaru T. (2001). Surgical strategy for spinal metastases. Spine.

[38] Barzilai O., Fisher C.G., Bilsky M.H. (2018). State of the Art Treatment of Spinal Metastatic Disease. Neurosurgery.

[39] Laufer I., Rubin D.G., Lis E., Cox B.W., Stubblefield M.D., Yamada Y., Bilsky M.H. (2013). The NOMS framework: Approach to the treatment of spinal metastatic tumors. Oncologist.

[40] Wald J.T. (2012). Imaging of spine neoplasm. Radiol. Clin. N. Am..

[41] Hur J., Yoon C.S., Ryu Y.H., Yun M.J., Suh J.S. (2008). Comparative study of fluorodeoxyglucose positron emission tomography and magnetic resonance imaging for the detection of spinal bone marrow infiltration in untreated patients with multiple myeloma. Acta Radiol..

[42] Ridley L.J., Han J., Ridley W.E., Xiang H. (2018). Winking owl sign: Unilateral absent pedicle. J. Med. Imaging Radiat. Oncol..

[43] Guillevin R., Vallee J.N., Lafitte F., Menuel C., Duverneuil N.M., Chiras J. (2007). Spine metastasis imaging: Review of the literature. J. Neuroradiol..

[44] Leake R.L., Mills M.K., Hanrahan C.J. (2019). Spinal Marrow Imaging: Clues to Disease. Radiol. Clin. N. Am..

[45] Pinter N.K., Pfiffner T.J., Mechtler L.L. (2016). Neuroimaging of spine tumors. Handb. Clin. Neurol..

[46] Shah L.M., Salzman K.L. (2011). Imaging of spinal metastatic disease. Int. J. Surg. Oncol..

[47] Liu M., Sequeiros R.B., Xu Y., He X., Zhu T., Li L., Lü Y., Huang J., Li C. (2015). MRI-guided percutaneous transpedicular biopsy of thoracic and lumbar spine using a 0.23t scanner with optical instrument tracking. J. Magn. Reson. Imaging.

[48] Santiago F.R., Kelekis A., Alvarez L.G., Filippiadis D.K. (2014). Interventional procedures of the spine. Semin Musculoskelet. Radiol..

[49] Akhtar I., Manucha V. (2019). Rapid On-site Evaluation of Spine Lesions. Neuroimaging Clin. N. Am..

[50] Nourbakhsh A., Grady J.J., Garges K.J. (2008). Percutaneous spine biopsy: A meta-analysis. J. Bone Jt. Surg. Am..

[51] Guzik G. (2015). Surgical Treatment in Patients with Spinal Tumors—Differences in Surgical Strategies and Malignancy-Associated Problems. An Analysis of 474 Patients. Ortop. Traumatol. Rehabil..

[52] Guo Q., Cui Y., Wang L., Lu X., Ni B. (2016). Single anterior approach for cervical spine fractures at C5-T1 complicating ankylosing spondylitis. Clin. Neurol. Neurosurg..

[53] Kumar N., Tan B., Zaw A.S., Khine H.E., Maharajan K., Lau L.L., Rajendran P.C., Gopinathan A. (2016). The role of preoperative vascular embolization in surgery for metastatic spinal tumours. Eur. Spine J..

[54] Yang B., Lu T., Li H. (2017). Single-Session Combined Anterior-Posterior Approach for Treatment of Ankylosing Spondylitis with Obvious Displaced Lower Cervical Spine Fractures and Dislocations. Biomed Res. Int..

[55] He A., Xie D., Cai X., Qu B., Kong Q., Xu C., Yang L., Chen X., Jia L. (2017). One-stage surgical treatment of cervical spine fracture-dislocation in patients with ankylosing spondylitis via the combined anterior-posterior approach. Medicine.

[56] Novegno F., Granaroli P., Ciccoritti L., Lunardi P., Fraioli M.F. (2019). Chylous fistula: Management of a rare complication following right anterior cervical spine approach. Eur. Spine J..

[57] Cole J.S., Patchell R.A. (2008). Metastatic epidural spinal cord compression. Lancet Neurol..

[58] Shan J., Jiang H., Ren D., Wang C. (2017). Anatomic Relationship Between Right Recurrent Laryngeal Nerve and Cervical Fascia and Its Application Significance in Anterior Cervical Spine Surgical Approach. Spine.

[59] Harel R., Stylianou P., Knoller N. (2016). Cervical Spine Surgery: Approach-Related Complications. World Neurosurg..

[60] Steinmetz M.P., Mekhail A., Benzel E.C. (2001). Management of metastatic tumors of the spine: Strategies and operative indications. Neurosurg. Focus.

[61] Cheung K.M., Mak K.C., Luk K.D. (2012). Anterior approach to cervical spine. Spine.

[62] Prezerakos G.K., Sayal P., Kourliouros A., Pericleous P., Ladas G., Casey A. (2018). Paravertebral tumours of the cervicothoracic junction extending into the mediastinum: Surgical strategies in a no man’s land. Eur. Spine J..

[63] Joubert C., Adetchessi T., Peltier E., Graillon T., Dufour H., Blondel B., Fuentes S. (2015). Corpectomy and Vertebral Body Reconstruction with Expandable Cage Placement and Osteosynthesis via the single stage Posterior Approach: A Retrospective Series of 34 Patients with Thoracic and Lumbar Spine Vertebral Body Tumors. World Neurosurg..

[64] Abduljabbar F.H., Teles A.R., Bokhari R., Weber M., Santaguida C. (2018). Laminectomy with or Without Fusion to Manage Degenerative Cervical Myelopathy. Neurosurg. Clin. N. Am..

[65] Chaichana K.L., Sciubba D.M., Li K.W., Gokaslan Z.L. (2009). Surgical management of thoracic spinal cord herniation: Technical consideration. J. Spinal Disord. Tech..

[66] Lubelski D., Abdullah K.G., Steinmetz M.P., Masters F., Benzel E.C., Mroz T.E., Shin J.H. (2013). Lateral extracavitary, costotransversectomy, and transthoracic thoracotomy approaches to the thoracic spine: Review of techniques and complications. J. Spinal Disord. Tech..

[67] Teng H., Xinghai Y., Wei H., Huang Q., Xiao J., Zhang C. (2011). Malignant fibrous histiocytoma of the spine: A series of 13 clinical case reports and review of 17 published cases. Spine.

[68] Howell E.P., Williamson T., Karikari I., Abd-El-Barr M., Erickson M., Goodwin M.L., Reynolds J., Sciubba D.M., Goodwin C.R. (2019). Total en bloc resection of primary and metastatic spine tumors. Ann. Transl. Med..

[69] Kato S., Demura S., Shinmura K., Yokogawa N., Yonezawa N., Shimizu T., Oku N., Kitagawa R., Murakami H., Kawahara N. (2020). Clinical outcomes and survivals after total en bloc spondylectomy for metastatic leiomyosarcoma in the spine. Eur. Spine J..

[70] Shimizu T., Murakami H., Sangsin A., Demura S., Kato S., Shinmura K., Yokogawa N., Oku N., Kitagawa R., Tsuchiya H. (2018). En bloc corpectomy for late gastrointestinal stromal tumor metastasis: A case report and review of the literature. J. Med. Case Rep..

[71] Guzik G. (2017). Outcomes of Corpectomy in Patients with Metastatic Cancer. Ortop. Traumatol. Rehabil..

[72] Meyer S.A., Singh H., Jenkins A.L. (2010). Surgical treatment of metastatic spinal tumors. Mt. Sinai J. Med..

[73] Holman P.J., Suki D., McCutcheon I., Wolinsky J.P., Rhines L.D., Gokaslan Z.L. (2005). Surgical management of metastatic disease of the lumbar spine: Experience with 139 patients. J. Neurosurg. Spine.

[74] Georgy B.A. (2008). Metastatic spinal lesions: State-of-the-art treatment options and future trends. AJNR Am. J. Neuroradiol..

[75] Boucher H.H. (1959). A method of spinal fusion. J. Bone Jt. Surg. Br..

[76] Vieweg U., van Roost D., Wolf H.K., Schyma C.A., Schramm J. (1999). Corrosion on an Internal Spinal Fixator System. Spine.

[77] Lonstein J.E., Denis F., Perra J.H., Pinto M.R., Smith M.D., Winter R.B. (1999). Complications associated with pedicle screws. J. Bone Jt. Surg. Am..

[78] Ringel F., Ryang Y.M., Kirschke J.S., Müller B.S., Wilkens J.J., Brodard J., Combs S.E., Meyer B. (2017). Radiolucent Carbon Fiber-Reinforced Pedicle Screws for Treatment of Spinal Tumors: Advantages for Radiation Planning and Follow-up Imaging. World Neurosurg..

[79] Facchini G., Di Tullio P., Battaglia M., Bartalena T., Tetta C., Errani C., Mavrogenis A.F., Rossi G. (2016). Palliative embolization for metastases of the spine. Eur. J. Orthop. Surg. Traumatol..

[80] Clausen C., Dahl B., Frevert S.C., Hansen L.V., Nielsen M.B., Lönn L. (2015). Preoperative embolization in surgical treatment of spinal metastases: Single-blind, randomized controlled clinical trial of efficacy in decreasing intraoperative blood loss. J. Vasc. Interv. Radiol..

[81] Geraets S.E.W., Bos P.K., van der Stok J. (2020). Preoperative embolization in surgical treatment of long bone metastasis: A systematic literature review. EFORT Open Rev..

[82] Houten J.K., Swiggett S.J., Hadid B., Choueka D.M., Kinon M.D., Buciuc R., Zumofen D.W. (2020). Neurologic Complications of Preoperative Embolization of Spinal Metastasis: A Systemic Review of the Literature Identifying Distinct Mechanisms of Injury. World Neurosurg..

[83] Winkler E.A., Rowland N.C., Yue J.K., Birk H., Ozpinar A., Tay B., Ames C.P., Mummaneni P.V., El-Sayed I.H. (2016). A Tunneled Subcricoid Approach for Anterior Cervical Spine Reoperation: Technical and Safety Results. World Neurosurg..

[84] Behnke N.K., Baker D.K., Xu S., Niemeier T.E., Watson S.L., Ponce B.A. (2017). Risk factors for same-admission mortality after pathologic fracture secondary to metastatic cancer. Support. Care Cancer.

[85] Aebi M. (2003). Spinal metastasis in the elderly. Eur. Spine J..

[86] Polly D.W., Chou D., Sembrano J.N., Ledonio C.G., Tomita K. (2009). An analysis of decision making and treatment in thoracolumbar metastases. Spine.

[87] Barzilai O., Robin A.M., O’Toole J.E., Laufer I. (2020). Minimally Invasive Surgery Strategies: Changing the Treatment of Spine Tumors. Neurosurg. Clin. N. Am..

[88] Lee C.Y., Wu M.H., Li Y.Y., Cheng C.C., Lee C.Y., Huang T.J. (2016). Video-Assisted Thoracoscopic Surgery and Minimal Access Spinal Surgery Compared in Anterior Thoracic or Thoracolumbar Junctional Spinal Reconstruction: A Case-Control Study and Review of the Literature. Biomed Res. Int..

[89] Huang T.J., Hsu R.W., Li Y.Y., Cheng C.C. (2006). Minimal access spinal surgery (MASS) in treating thoracic spine metastasis. Spine.

[90] Gu Y., Dong J., Jiang X., Wang Y. (2016). Minimally Invasive Pedicle Screws Fixation and Percutaneous Vertebroplasty for the Surgical Treatment of Thoracic Metastatic Tumors with Neurologic Compression. Spine.

[91] Barzilai O., DiStefano N., Lis E., Yamada Y., Lovelock D.M., Fontanella A.N., Bilsky M.H., Laufer I. (2018). Safety and utility of kyphoplasty prior to spine stereotactic radiosurgery for metastatic tumors: A clinical and dosimetric analysis. J. Neurosurg. Spine.

[92] Yahanda A.T., Buchowski J.M., Wegner A.M. (2019). Treatment, complications, and outcomes of metastatic disease of the spine: From Patchell to PROMIS. Ann. Transl. Med..

[93] Ozdemir Y., Torun N., Guler O.C., Yildirim B.A., Besen A.A., Yetisken A.G., Onal H.C., Topkan E. (2019). Local control and vertebral compression fractures following stereotactic body radiotherapy for spine metastases. J. Bone Oncol..

[94] Al Farii H., Frazer A., Farahdel L., Alfayez S., Weber M. (2020). Zoledronic Acid Versus Denosumab for Prevention of Spinal Cord Compression in Advanced Cancers with Spine Metastasis: A Meta-Analysis of Randomized Controlled Trials. Glob. Spine J..

[95] Benjamin R. (2002). Neurologic complications of prostate cancer. Am. Fam. Physician.

[96] Dong P., Chen N., Li L., Huang R. (2017). An upper cervical cord compression secondary to occult follicular thyroid carcinoma metastases successfully treated with multiple radioiodine therapies: A clinical case report. Medicine.

[97] Diaby V., Tawk R., Sanogo V., Xiao H., Montero A.J. (2015). A review of systematic reviews of the cost-effectiveness of hormone therapy, chemotherapy, and targeted therapy for breast cancer. Breast Cancer Res. Treat..

[98] Nader R., El Amm J., Aragon-Ching J.B. (2018). Role of chemotherapy in prostate cancer. Asian J. Androl..

[99] Coleman R., Body J.J., Aapro M., Hadji P., Herrstedt J., ESMO Guidelines Working Group (2014). Bone health in cancer patients: ESMO Clinical Practice Guidelines. Ann. Oncol..

[100] Park J.S., Park S.J., Lee C.S. (2010). Incidence and prognosis of patients with spinal metastasis as the initial manifestation of malignancy: Analysis of 338 patients undergoing surgical treatment. Bone Jt. J..

[101] Ejima Y., Matsuo Y., Sasaki R. (2015). The current status and future of radiotherapy for spinal bone metastases. J. Orthop. Sci..

[102] Kropczyński G., Gabriel A., Kusz D., Ryś J., Miszczyk L., Paściak M. (2009). Influence of the type of neoplasm and treatment on the survival of patients with malignant spinal tumours. Ortop. Traumatol. Rehabil..

[103] Oh M.C., Kim J.M., Kaur G., Safaee M., Sun M.Z., Singh A., Aranda D., Molinaro A.M., Parsa A.T. (2013). Prognosis by tumor location in adults with spinal ependymomas. J. Neurosurg. Spine.

[104] Gilbert M.R., Ruda R., Soffietti R. (2010). Ependymomas in adults. Curr. Neurol. Neurosci. Rep..

